# Utility of HoxB13 in differential diagnosis of female genital tract lesions with putative prostatic differentiation

**DOI:** 10.1007/s00428-025-04360-7

**Published:** 2025-12-03

**Authors:** Lucie Gerykova, Jan Laco, Helena Hornychova, Katerina Kamaradova, Marketa Trnkova, Pavel Dundr, Radoslav Matej, Jan Hrudka, Miroslav Zalesky, Jiri Soukup

**Affiliations:** 1 The Fingerland Department of Pathology, Charles University, Faculty of Medicine in Hradec Králové and University Hospital Hradec Králové, Sokolska 581, Hradec Kralove, 500 05 Czech Republic; 2 Unilabs Pathology K.S., Evropska 2589/33B, Praha 6, Prague, 160 00 Czech Republic; 3 Department of Pathology, Charles University, First Faculty of Medicine and General University Hospital in Prague, Studnickova 2039, Nové Mesto, Prague, 128 00 Czech Republic; 4 Department of Pathology, Third Faculty of Medicine, Charles University, University Hospital Královské Vinohrady, Srobarova 50, Praha 10, Prague, 100 34 Czech Republic; 5 Department of Pathology, Military University Hospital Prague, U Vojenske Nemocnice 1200, Praha 6, Prague, 169 02 Czech Republic; 6 Department of Pathology and Molecular Medicine, Third Faculty of Medicine, Charles University, Thomayer University Hospital, Videnska 800, Praha 4, Prague, 140 59 Czech Republic; 7 Department of Urology, First Faculty of Medicine, Charles University and Military University Hospital Prague, Prague, Czech Republic

**Keywords:** Adenoid basal carcinoma, Tubulosquamous polyp, Cervix, NKX3.1, HoxB13

## Abstract

Female genital tract lesions with putative prostatic differentiation include vaginal tubulosquamous polyp (TSP), cervical ectopic prostatic tissue (EPT), and adenoid basal carcinoma (ABC). HoxB13 is a transcription factor specific for the prostate, not previously assessed in these lesions. A cohort of 13 TSPs, 6 EPTs, 17 ABCs, and 8 adenoid basal hyperplasia (ABH) was analyzed for expression of prostatic markers HoxB13, NKX3.1, p16, and androgen receptor (AR). Additional 28 cervical high-grade squamous intraepithelial lesions (HSILs), 21 cervical squamous cell carcinomas (SCCs), 19 endocervical adenocarcinomas (EACs), and 10 endometrial endometrioid adenocarcinomas (ECs) were included. The results were recorded as immunoreactive score (IRS). In TSPs and EPTs, HoxB13 and NKX3.1 were positive in 100% and 89.5% of cases, respectively. No HoxB13, NKX3.1, and p16 was observed in ABHs. In contrast, all ABCs showed diffuse p16 positivity. NKX3.1 was positive in 82.6% of ABCs (median IRS = 2), HoxB13 was positive in 100% of ABCs (median IRS = 8), and the difference was statistically significant (p < 0.001). Both HoxB13 and NKX3.1 were positive in 9.5% (2/21) SCCs and 21.1% (4/19) EACs. Additionally, 40% (4/10) ECs were HoxB13-positive (IRS = 1–2). Any positivity of HoxB13 was 80% specific and 100% sensitive for the ABC. NKX3.1 was only 82.4% sensitive, but 88% specific for the diagnosis of ABC. Additionally, HoxB13 was also seen in 29.8% HSILs. No ABC showed AR positivity, while this was observed in rare cells (IRS 1–2) in 30.8%, 16.6%, and 37.5% of TSCs, EPTs, and ABHs, respectively. HoxB13 can be a useful and reliable marker for identification of TSPs, EPTs and ABCs.

**Supplementary Information:**

The online version contains supplementary material available at 10.1007/s00428-025-04360-7.

## Introduction

Developmental analogies exist between structures of male and female lower genitourinary tract. At the end of third month of embryonal development, proliferation of the urethral epithelium gives rise to protrusions placed in the periurethral mesenchyme [1]. In males, prostatic gland is formed from these protrusions, while two paraurethral (Skene’s) glands develop in females [1, 2]. These are considered a female analogue of male prostate [3]. Interestingly, two lesions occasionally occurring in vagina and cervix – tubulosquamous polyp (TSP) and ectopic prostatic tissue (EPT)—are considered a derivative of the Skene´s gland and express selected prostatic markers [3]. Additional studies propose that adenoid basal carcinoma (ABC) of the cervix may also share prostatic differentiation, as suggested by the common immunoreactivity of prostate specific proteins such as prostate specific antigen or NKX3.1 [3, 4].

NKX3.1, androgen receptor (AR) and HoxB13 are often used as markers of prostatic differentiation. HoxB13 is a member of homeobox (HOX) family of transcription factors encoded by *HOXB13* localized on chromosome 17 [5]. It confers spatial identity of embryonal tailbud, with expression in caudal domains of the spinal cord, digestive tract, and urogenital sinus [5, 6]. With other caudal HOX genes, *HOXB13* is instrumental in normal prostate development, and the gene expression remains high during post-embryonal period, with preferential expression in prostatic luminal cells [6, 7]. The prostatic HoxB13 expression seems to be androgen independent, in contrast to NKX3.1 [6, 7]. NKX3.1 is a homeobox-containing transcription factor encoded by *NKX3.1* gene localized on chromosome 8 (20). It is predominantly expressed in luminal prostatic epithelium [8] and plays an important role in its differentiation [9]. HoxB13 functions as a transcriptional coactivator for multiple genes important in establishing prostate cell identity, including NKX3.1 whose expression is otherwise AR-dependent [10]. Although HoxB13 expression is observed mainly in luminal cells, basal cell population may also show a weak immunoreactivity [6], in contrast to NKX3.1 which is reportedly lacking in prostatic basal cells [8].

Given its role in normal prostate, immunohistochemical detection of NKX3.1 is commonly used as a marker of prostatic origin [8, 11–13]. However, NKX3.1 is not entirely specific for prostatic tissue identity as it may be observed in a subset of breast carcinomas [13, 14] and salivary gland tumors [15, 16] . In the past, HoxB13 was also described as a highly sensitive and specific marker of prostatic carcinoma [17], although additional reports yielded conflicting results with respect to its diagnostic utility, compared to other prostatic markers [12, 18, 19].

Since HoxB13 provides developmental identity to prostatic cells, we decided to analyze its expression in lesions of female genital tract with presumed prostatic differentiation (TSP, EPT and ABC). We were additionally interested in prostatic phenotype of adenoid basal hyperplasia (ABH), a lesion that morphologically resembles ABC but lacks deep stromal invasion [20]. Although it is considered distinct to ABC, its “prostatic” features has not been documented previously. Since the expression of currently used prostatic markers (NKX.3.1, PSA, PSAP) in TSPs and EPTs can be limited to the glandular cells, we postulated that given its occasional presence in basal cells and androgen independent regulation, HoxB13 may stand the test as a more robust diagnostic marker. We analyzed a large cohort of ABCs, ABHs, TSPs, and EPTs for immunohistochemical expression of HoxB13, NKX3.1 and AR. Additionally, we assessed immunoreactivity of HoxB13 and NKX3.1 in a cohort of cervical high grade squamous intraepithelial lesions (HSILs), cervical squamous cell carcinomas (SCCs), endocervical adenocarcinomas (EACs) and high-grade endometrial endometrioid adenocarcinomas (ECs).

## Materials and methods

The archives of all the authors were searched for cases diagnosed as TSP, EPT, ABH, ABC. All the cases were reviewed by an experienced gynecopathologist (JL) prior to the inclusion in the study, in order to confirm the diagnosis. Additional cases of HSILs, SCCs, EACs, and ECs, as well as prostate samples with basal cell hyperplasia were retrieved from the archive and reviewed by an experienced surgical pathologist (JL or JS). Cases of ECs were preferentially selected to include tumors with endocervical invasion (n = 5). The study was conducted in accordance with the Declaration of Helsinki, and the ethical committee of the first author’s institution approved the study.

The formalin fixed paraffin embedded tissue blocks were cut into 3 μm-thick tissue sections for immunohistochemical analysis. Heat induced epitope retrieval (HIER) was used for antigen retrieval according to the antibody. The staining of the NKX3.1 (EP356, 1:100, Cell Marque, Rocklin, CA, USA), AR (AR411, 1:40, Dako, Glostrup, Denmark), and p16 (R15-A, 1:100, DB Biotech, Kosice, Slovakia) was carried out on automated platform Agilent/Dako Omnis (Agilent, Santa Clara, CA, USA). HoxB13 (D7N8O, 1:500, Cell Signaling, Danvers, MA, USA) detection was performed on Benchmark Ultra (Ventana/Roche, Tucson, AZ, USA). Visualization used polymer systems (Ventana OptiView or Dako EnVision Flex) with horseradish peroxidase as enzyme and DAB (3,3’-diaminobenzidine) as chromogen. All slides were counterstained with hematoxylin and a prostate tissue (HoxB13, NKX3.1) as a positive control was included on each slide. The analysis of NKX3.1 and HoxB13 was performed in all the cases. AR immunohistochemistry was performed only in lesions with putative prostatic differentiation (TSP, EPT, ABH, ABC). Detection of p16 was performed only in ABCs and ABHs. In one ABC and two EPTs, not enough material was available and thus, archival slides (ABC – p16; EPTs—NKX3.1) were analyzed instead.

All immunohistochemical markers were evaluated by 2 pathologists independently (JS and LG). p16 was scored as either positive (strong block positivity in > 70% of the lesion) or negative. Results of NKX3.1, HoxB13, and AR were evaluated as a percentage of positive cells and staining intensity (1 +, 2 +, 3 +). In lesions with both squamous and glandular differentiation (TSPs, EPTs and ABCs), the lesion was first evaluated as a whole, and glandular structures were then scored separately. Features of HSILs associated with ABCs were also scored and recorded separately. The final percentage of positive cells was established as the average of both observations. The cases with discordant intensity were discussed at the double-headed microscope for consensus. Immunoreactive score (IRS) according to Remmele [21] was established as a product of intensity (1–3) and overall percentage of all positive cells (0 – no positive cells; 1—< 10%; 2–11–50%; 3–51–80%; 4—> 80%), spanning between 1 and 12 in positive cases. All detailed clinical and morphological data alongside with IRS scores generated in the study are available in Supplementary Table 1.

Statistical analysis was performed using GraphPad Prism 8.0.1 software (GraphPad Software, Inc., USA). D’Agostino-Pearson normality test was used to assess the distribution of the data. In all cases, parametric values were described with mean and standard deviation (SD); nonparametric values were described with median and interquartile range (IRQ). Wilcoxon matched pairs signed rank test (WT) and X^2^ test were used. P-values with Bonferroni correction for two tested markers (HoxB13 and NKX3.1) < 0.025 were considered statistically significant.

## Results

### Clinical and pathological characteristics of TSP and EPT

**Fig. 1:**
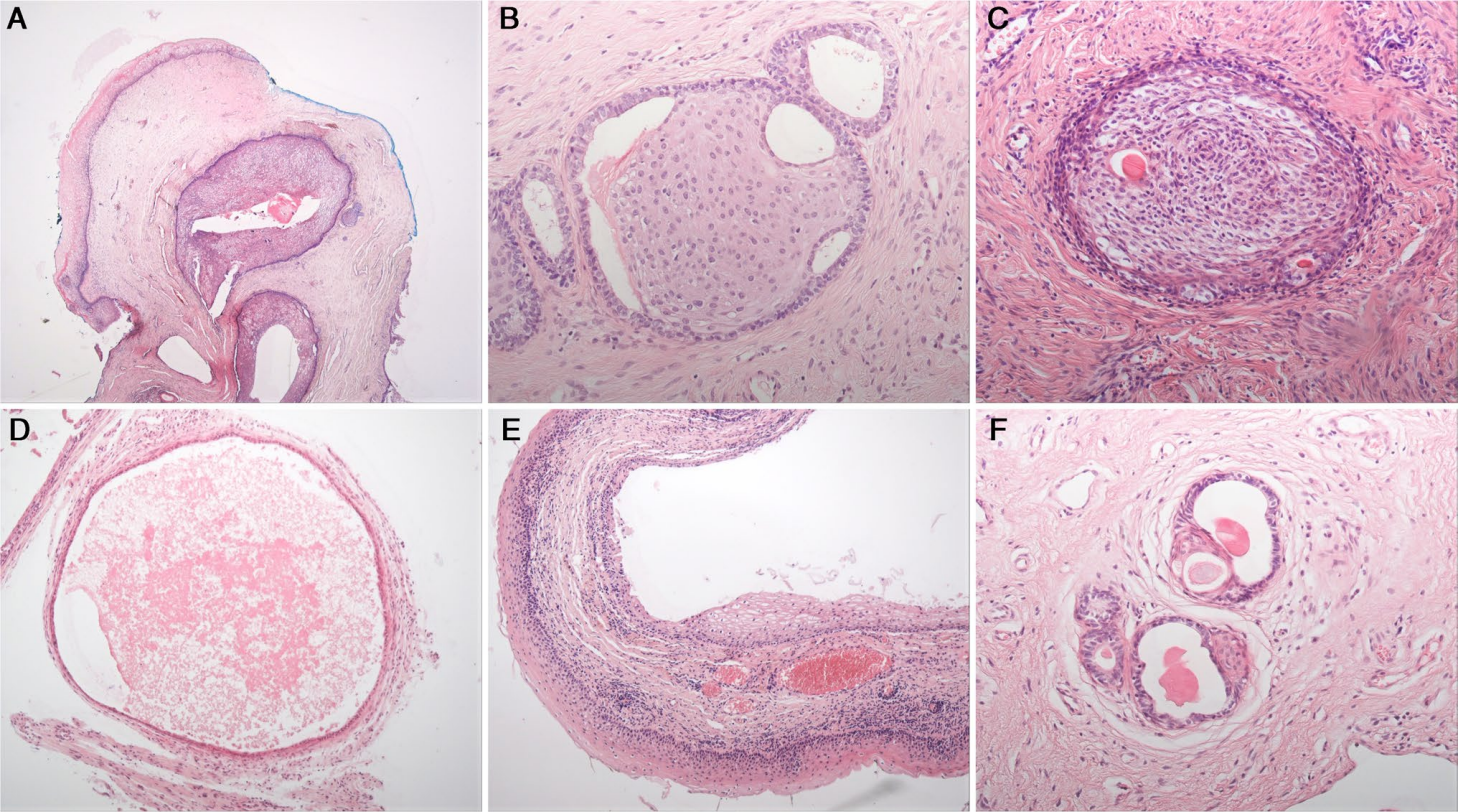
Morphological features of TSP: (**A**) Polypoid configuration of TSP (original magnification 20x, H&E). (**B**) Numerous tubules present in one case of TSP (original magnification 200x, H&E). (**C**) Only a small number of tubules present in one case of TSP (original magnification 200x, H&E). (**D**) A tubule showing prominent dilatation (original magnification 100x, H&E). (**E**) A case of TSP without identifiable tubular structures (original magnification 100x, H&E). (**F**) TSP consisting predominantly of tubular structures (original magnification 200x, H&E)

**Fig. 2:**
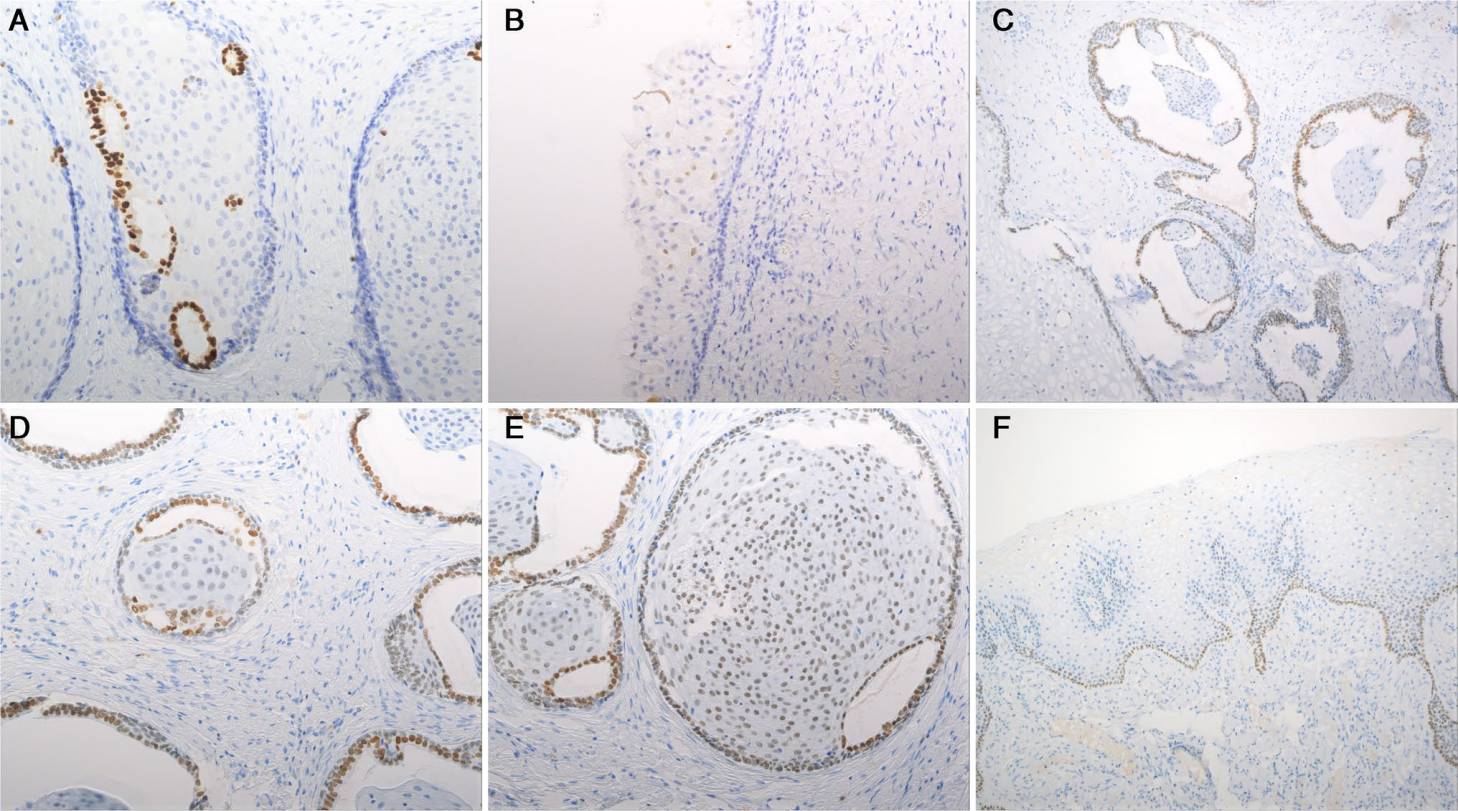
Immunoprofile of TSP: (**A**) Positive expression of NKX3.1 in tubular structures of TSP (original magnification 200x, H&E) (**B**) A small number of cells of squamous nests with a weak expression of NKX3.1 (original magnification 200x, H&E) (**C**) Expression of HoxB13 in TSP (original magnification 100x, H&E) (**D**) Expression of HoxB13 in tubular structures of TSP (original magnification 200x, H&E) (**E**) Expression of HoxB13 in cells of squamous nests of TSP (original magnification 200x, H&E) (**F**) Expression of HoxB13 in the basal layer of the surface squamous epithelium (original magnification 100x, H&E)

The cohort included 13 TSPs and 6 EPTs. TSPs manifested clinically as a mass of the vaginal wall in 12 cases with available clinical data. Median age of the patients was 66 years, ranging from 51 to 86 years. On the other hand, 5 out of 6 EPTs were an incidental finding in cervical conization samples for HSIL. Median age of the patients was 48 years, ranging between 24 and 71 years. All the TSPs showed polypoid configuration (Fig. 1A), except for one highly fragmented case. The lesions were localized underneath vaginal epithelium and in 11 cases, they consisted of multiple squamous nests that contained variable number of tubular structures, ranging from numerous to sparse (Fig. 1B, C). In general, tubules were mainly small, difficult to identify, although in 3 cases, tubules showed prominent dilatation (Fig. 1D). One case lacked identifiable tubular structures (Fig. 1E), while in another, tubular structures largely predominated over squamous nests (Fig. 1F). In total, 30.8% (4/13) of the cases were AR-positive (IRS 1, 1, 2, and 2), while tubular structures were positive only in 16.6% (2/12) TSPs (both IRS 2). NKX3.1 was observed in 84.6% (11/13, median IRS 3) of TSPs, with strong predilection for tubular structures (91,7%, 11/12, median IRS 12, Fig. 2A), although some immunoreactivity was also observed in squamous nests in 3 cases (Fig. 2B). Finally, HoxB13 was observed in 100% (13/13, median IRS 2, Fig. 2C) of TSPs, being detectable in tubules in 90% (9/10, median IRS 7, Fig. 2D) of the cases. In contrast to NKX3.1, HoxB13 was also commonly observed in squamous cells, showing overall immunoreactivity in 84.6% (11/13, Fig. 2E) of the cases. Interestingly, HoxB13 was seen in the basal layer of the overlying vaginal epithelium in 69.2% (9/11, Fig. 2F) of the cases.

**Fig. 3:**
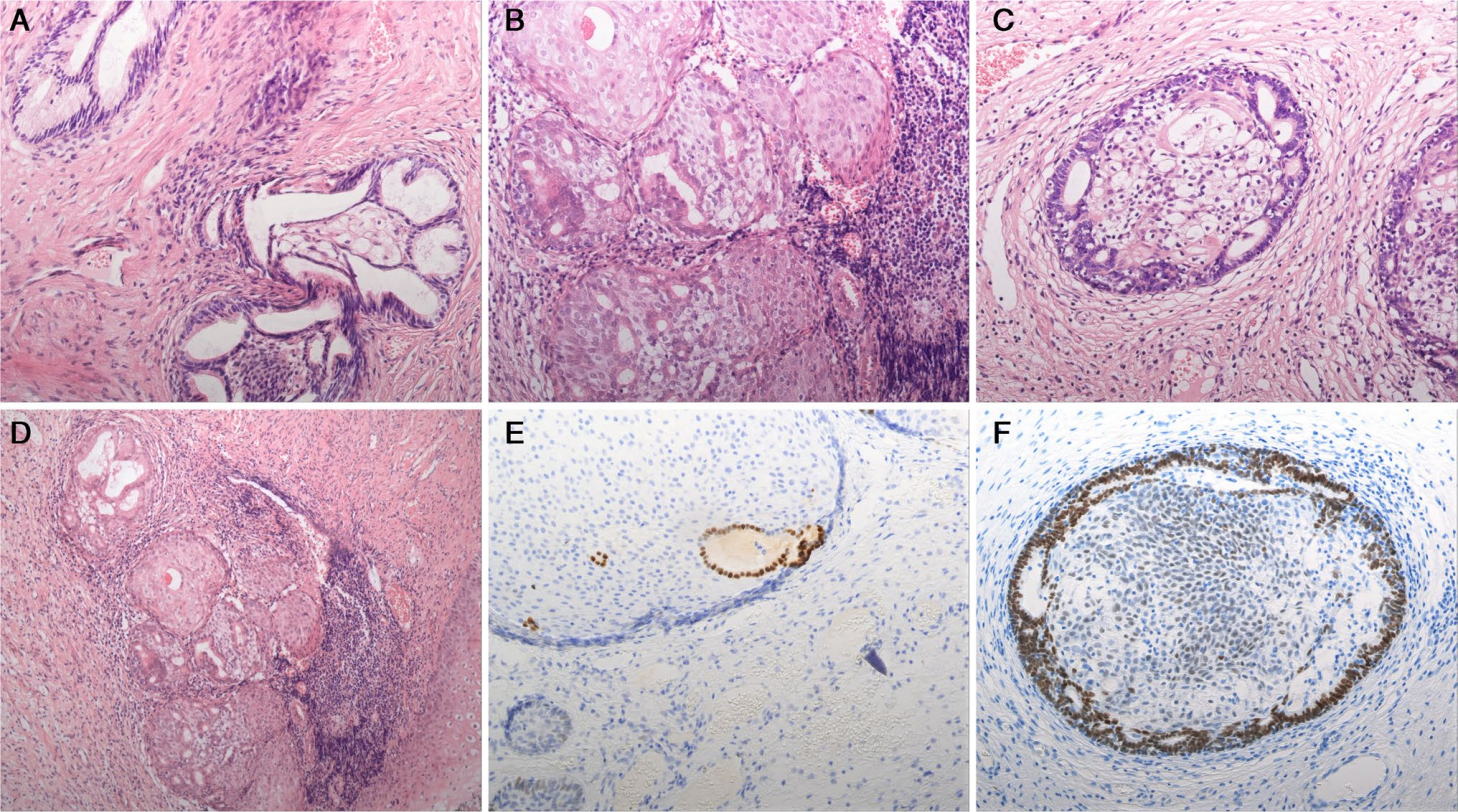
Morphological features and immunoprofile of EPT: (**A**) Prostatic-like glands in EPT (original magnification 200x, H&E). (**B**) Squamous nests ensheathing the glands (original magnification 200x, H&E). (**C**) Squamous nests containing small tubules inside them (original magnification 200x, H&E). (**D**) A case of EPT with combination of small tubules and prostatic like glands (original magnification 200x, H&E). (**E**) Expression of NKX3.1 in glandular structures of EPT (original magnification 200x, H&E). (**F**) Expression of HoxB13 in tubules and squamous cells of EPT (original magnification 200x, H&E)

The EPT consisted of glands resembling the prostatic gland in 50% (3/6, Fig. 3A). In one case, squamous nests ensheathed the glands (Fig. 3B) and in additional two EPTs, the overall appearance resembled TSP (Fig. 3C). In one case, a combination of small tubules within squamous nests and “prostatic-like” glands was seen (Fig. 3D). AR was positive in 16.6% (1/6, IRS 2), NKX3.1 was seen in 100% (6/6, median IRS 6 for all the cells and IRS 12 for glandular structures, Fig. 3E), and HoxB13 was also positive in 100% (5/5) of the EPTs (median IRS 4 for all the cells and IRS 8 for glandular structures, Fig. 3F). HoxB13 was more commonly observed in squamous areas, compared to NKX3.1 (4 cases vs 1 case).

Comparing IRS scores of NKX3.1 and HoxB13 in TSPs and EPTs, we observed no significant differences between scores of whole lesions (p = 0.73, WT). However, NKX3.1 was significantly stronger in tubular structures, compared to HoxB13 (p < 0.001, WT). The results of the immunohistochemistry in individual subsets of the cohort are presented in Table 1.

**Table 1:** The results of the immunohistochemistry in individual subsets of the cohort

	NKX3.1 positivity rate	HoxB13 positivity rate	NKX3.1 median IRS (IQR)	HoxB13 median IRS (IQR)
Tubulosquamous polyps (*n* = 13)	84.6%	100%	3 (1–4.5)	2 (2–4)
Ectopic prostatic tissue	100% (*n* = 6)	100% (*n* = 5)	6 (3.75–9.75)	4 (3–9)
Adenoid basal hyperplasia	0 (*n* = 8)	0 (*n* = 6)	0	0
Adenoid basal carcinoma	82.6% (*n* = 17)	100% (*n* = 15)	2 (1–6)	8 (4–12)
High grade squamous intraepithelial lesion (*n* = 28)	10.7%	50%	0 (0–0)	0.5 (0–2)
Squamous cell carcinoma (*n* = 21)	9.5%	9.5%	0 (0–0)	0 (0–0)
Endocervical adenocarcinoma (*n* = 19)	21.1%	21.1%	0 (0–0)	0 (0–0)
Endometroid carcinoma (*n* = 10)	0	40%	0	0 (0–1.25)

### Clinical and pathological characteristics of ABH

**Fig. 4:**
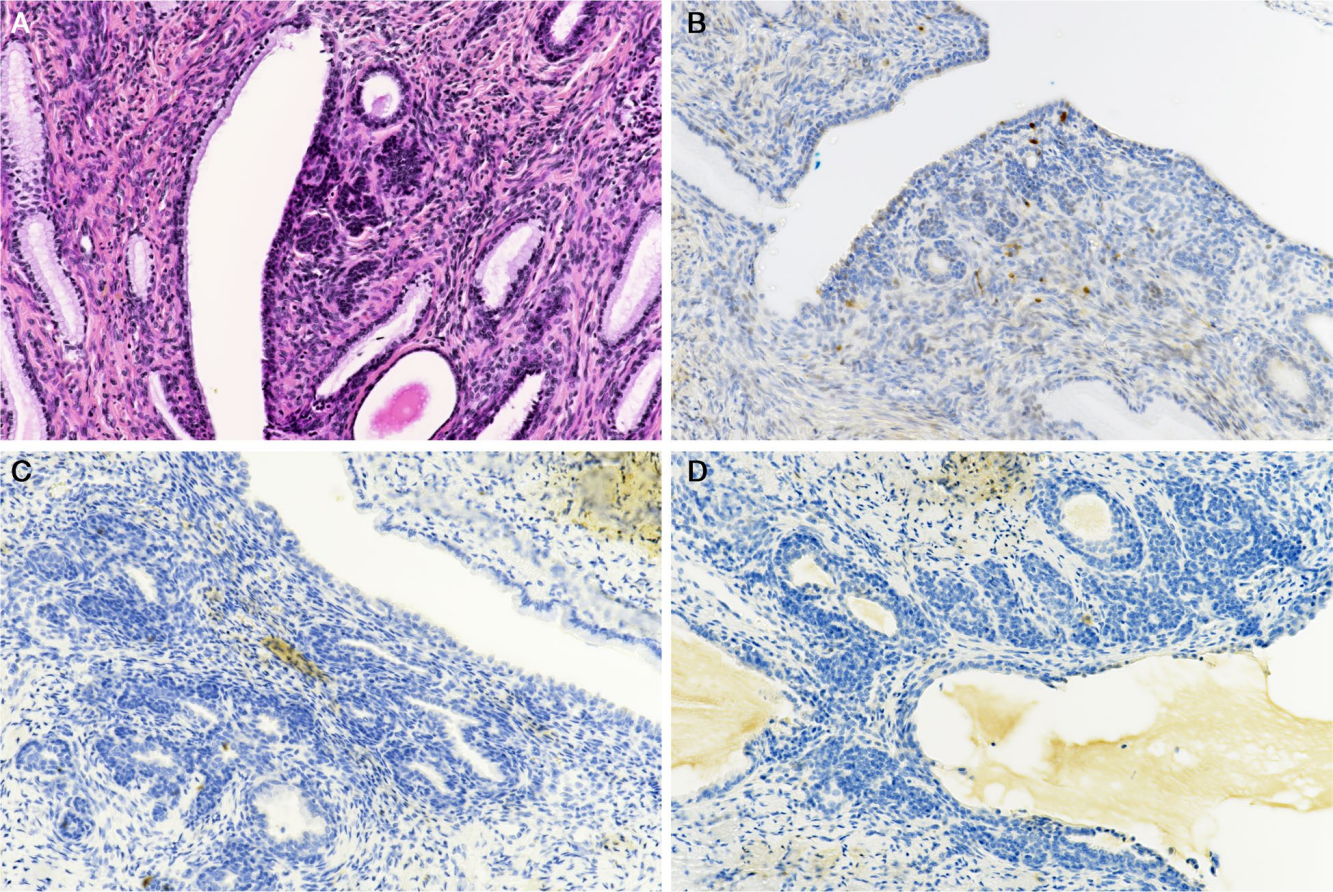
Morphological features and immunoprofile of ABH: (**A**) Small basaloid nests without identifiable tubular structures inside them. (**B**) Negative expression of p16 in ABH. (**C**) Negative expression of NKX3.1 in ABH. (**D**) Negative expression of HoxB13 in ABH

All 8 cases of ABH included in the study were incidental findings in hysterectomy or cervical conization samples. In all the cases, small basaloid nests extending less than 1 mm from the basement membrane were seen surrounding the endocervical canal, with no identifiable tubular structures (Fig. 4A). The ABHs were negative for p16 (n = 8, Fig. 4B), NKX3.1 (n = 8, Fig. 4C) and HoxB13 (n = 6, Fig. 4D). In 37.5% (3/8) of the cases, weak AR positivity (IRS scores 1, 2, and 2) were observed. ABH was associated with HSIL in 2 cases and in 1 case, with ABC.

### Clinical and pathological characteristics of ABC

**Fig. 5:**
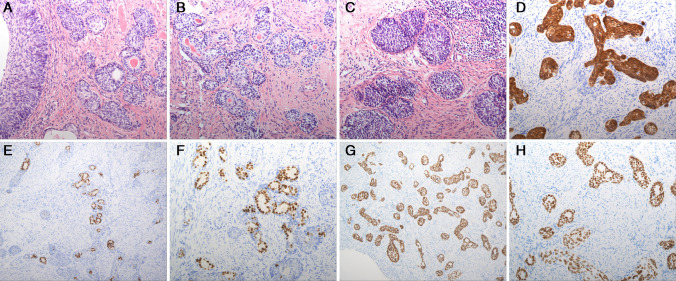
Morphological features and immunoprofile of ABC: (**A**) HSIL (on the left) present alongside with ABC (on the right, original magnification 200x, H&E). (**B**) Numerous small glandular structures present in ABC (original magnification 200x, H&E). (**C**) No glandular structures present in one case of ABC (original magnification 200x, H&E). (**D**) Diffuse block expression of p16 (original magnification 200x, H&E). (**E**) Immunohistochemical expression of NKX3.1 in ABC (original magnification 100x, H&E). (**F**) Immunohistochemical expression of NKX3.1 in glandular structures of ABC (original magnification 200x, H&E). (**G**) Immunohistochemical expression of HoxB13 in ABC (original magnification 100x, H&E). (**H**) Immunohistochemical expression of HoxB13 in glandular structures as well as in basaloid cells of the nests of ABC (original magnification 200x, H&E)

The study included 17 ABCs. The median age at the diagnosis was 67 years (range 27–75 years). In 2 cases, ABC was an incidental finding in hysterectomy performed for non-cervical pathology. HSIL was the reason of the surgery in 14 cases and invasive SCC in 2 cases. HSIL associated with ABC was present in 76.5% (13/17) of the cases (Fig. 5A).

All the ABCs consisted of small to moderately sized nests made of basophilic cells with small nuclei and high N/C ratio. In 10 cases, predominantly smaller tubular/glandular structures were seen within the nests of ABC (Fig. 5B). In three cases, no tubule formation was observed (Fig. 5C). All the ABCs were p16 positive (Fig. 5D). We observed no immunoreactivity of AR in any of ABCs. NKX3.1 and HoxB13 could be analyzed in 17 and 15 ABCs, respectively. NKX3.1 expression was observed in 82.6% (14/17, median IRS 2, Fig. 5E) of the cases and the majority of immunoreactive cells were present in glandular areas (92.9%, 13/14, median IRS 8, Fig. 5F), while only rare basaloid cells showed NKX3.1 positivity. In contrast, HoxB13 was positive in 100% of ABCs (median IRS 8, Fig. 5G), with strong positivity in 100% of tubular areas (13/13, median IRS 12, Fig. 5H). Overall lesion immunoreactivity for HoxB13 was significantly stronger compared to NKX3.1 (median IRS 8 vs 2, p < 0.001, WT), although no difference was observed in immunoreactivity of glandular structures (p = 0.33, WT).

### Immunoreactivity of HoxB13 and NKX3.1 in other cervical carcinomas

We additionally analyzed 21 SCCs (all p16-positive), 19 EACs (17 HPV-associated usual type, 1 HPV-associated mucinous type (signet-ring cell), 1 HPV-independent gastric type), and 10 ECs (n = 10, all FIGO grade 3 (high-grade) with cervical stromal invasion in 5 tumors) for NKX3.1 and HoxB13. In SCCs, HoxB13 was observed in 9.5% (2/21, IRSs 8 and 1) and NKX3.1 was also seen in 9.5% (2/21, IRSs 1 in both). In EACs, HoxB13 was observed in 21.1% (4/19, IRSs 1, 2, 2, and 4). Expression of NKX3.1 was also observed in 21.1% (4/19, IRSs 1, 1, 2, and 2). Two HPV-associated EACs (usual type and mucinous type (signet-ring cell)) expressed both HoxB13 and NKX3.1. As for grade 3 ECs, 40% (4/10) showed HoxB13 immunoreactivity (IRS scores 1, 1, 2 and 2) while they were all negative for NKX3.1. Additionally, we evaluated sensitivity and specificity of both markers for the ABC in the differential diagnosis of invasive cervical carcinomas. Any positivity of HoxB13 was 80% specific and 100% sensitive for the ABC. On the other hand, NKX3.1 was only 82.4% sensitive, but 88% specific for the diagnosis of ABC.

### Expression of HoxB13 and NKX3.1 in HSIL

In total, HSIL was present in 28 cases out of 94 cases included in the study. As for HoxB13, we identified predominantly weak immunoreactivity (median IRS 2, IRS range 1–9) in 50% (14/28) of cases. 10 positive cases were associated with ABC, 1 case with SCC (the associated carcinoma was HoxB13 negative), and 3 cases with EACs. Of these, 2 cases of EACs also expressed HoxB13. HSIL immunoreactivity was observed in 90.9% (10/11) of ABC-associated HSILs, but only in 23.5% (4/17) of ABC-unrelated HSILs and this association was statistically significant (p < 0.001, X^2^). NKX3.1 expression was found in 10.7% (3/28) of HSILs (IRS scores 1, 1, 4). One case was associated with ABC, 1 case with HoxB13-negative SCC and 1 case with HoxB13-positive signet ring cell mucinous EAC.

### Expression of HoxB13 and NKX3.1 in normal tissues

In 36 cases of the cohort, expression of HoxB13 was observed in the basal layer of the surface squamous epithelium (in 9 cases of TSP, 4 cases of EPT, 8 cases of ABC, 6 cases of SCC, 8 cases of EAC, 1 case of EC). Additionally, we assessed HoxB13 in 5 prostate samples with basal cell hyperplasia. All selected cases strongly expressed HoxB13 in the luminal cell of the prostatic glands, while basal cells decorated weakly with the antibody (all positive, IRS ranging 2–3). On the other hand, NKX3.1 was not identified in surrounding normal tissues in any of the studied case.

## Discussion

Our study focused on expression of NKX3.1, HoxB13 and AR in lesions of female genitourinary tract with possible prostatic differentiation. These include tubulosquamous polyps of vagina (TSP), ectopic prostatic tissue in the cervix (EPT), and adenoid basal carcinoma (ABC) of the cervix. NKX3.1 and other prostatic markers (PSA, PSAP) have been studied previously [3, 4] in these lesions, however, these markers are not entirely specific for the prostatic differentiation. NKX3.1 can be expressed in salivary gland or male breast tumors, usually in androgen receptor-positive tumors, as the expression of the transcription factor is generally androgen dependent [9, 10]. A subset of soft tissue sarcomas is also NKX3.1 positive, that might be related to NKX3.1 expression in paraxial mesoderm of the developing embryo [9]. Similarly, PSA and PSAP can be observed in salivary gland tumors and a subset of well differentiated neuroendocrine tumors. On the other hand, HoxB13 expression is androgen independent and confined to caudal parts of the embryo during the development, including urogenital sinus [5, 6]. Up to this day, HoxB13 was observed only in tumors of the prostate [6, 17], rectal well differentiated neuroendocrine tumors [22], colorectal adenocarcinomas [6] and neuroectodermal tumors of caudal spine, including cauda equina neuroendocrine tumors [23] and myxopapillary ependymomas [24]. HoxB13 immunohistochemistry seems highly sensitive and specific for these tumors, especially using the novel clone D7N8O [6], in contrast to previously utilized F-9 clone [12]. However, NKX3.1 is widely used in diagnostic pathology at the moment, with established recommendations for the assay calibration (i.e. NordiQC recommendation). This makes NKX3.1 more standardized and available at the moment, although the role of HOXB13 in diagnostic neuropathology [24] and other fields is currently increasing [6, 22].

First, we assessed NKX3.1 and HoxB13 in EPTs and TSPs. Both entities probably arise from Skene´s glands and hence would be expected to show prostatic immunoprofile. Indeed, we observed expression of HoxB13 in all EPTs and TSPs, while NKX3.1 was observed in 89.5% (17/19) of the cases. While we did not find any significant difference in expression of NKX3.1 and HoxB13 when TSPs and EPTs were evaluated as a whole lesion, we found a statistically significant difference when expression of both markers was evaluated on glandular structures only. Our findings imply that while NKX3.1 and HoxB13 are both suitable for the diagnosis of TSP and EPT, NKX3.1 seem to be superior to HoxB13 for detection of the glandular structures.

In the second part or the study we focused on ABC and its potential mimics, including ABH. ABH is usually composed of a proliferation of basaloid cells attached to the surface cervical epithelium in the area of squamocolumnar junction. The lesion forms buds and small nests and characteristically lack involvement of deeper portions of the stroma [20]. It is occasionally observed adjacent to other cervical lesions, including ABC, and it shares morphological resemblance to ABC. The only available comparative study of ABCs and ABHs did not demonstrate high risk human papillomavirus (HR-HPV) infection and p16 immunoreactivity in any of 10 ABHs [20]. In contrast, 81.8% (9/11) of ABCs were p16 positive or HR-HPV associated [4, 20]. Interestingly, Goyal et al. [25] analyzed 17 cases of adenoid basal tumors of the cervix and showed lack of p16 in all 11 lesions designated as low-grade adenoid basal tumors. These were characterized by the lack of stromal invasion, mitotic activity or cellular atypia, and benign course on limited follow-up [25]. Given the clinicopathological description, it seems that these “low-grade adenoid basal tumors” were in fact ABHs. In our cohort, none of 8 ABHs was p16 positive, in contrast to strong and diffuse immunoreactivity of p16 in all 17 ABCs. Vast majority of our ABCs also showed associated HSIL or invasive squamous carcinoma. In contrast, associated HSIL was observed in 37.5% (3/8) ABHs and only one case was associated with ABC. Mostly, ABHs were incidental findings in a surgery sample for unrelated pathology. Additionally, HoxB13 and NKX3.1 were consistently negative in all ABHs, in contrast to ABCs (see below). Taken together, our findings support unrelated nature of both entities and provide useful diagnostic markers (p16, HoxB13 and NKX3.1) to distinguish them.

Finally, we assessed HoxB13 and NKX3.1 in ABCs, HSILs and other carcinomas of the cervix. NKX3.1 was positive in 82.6% and HoxB13 in 100% of ABCs. HoxB13 immunoreactivity was significantly stronger (median IRS 8 vs IRS 2), due to the more widespread immunoreactivity of basaloid cells. On the other hand, NKX3.1 showed predominantly glandular pattern of positivity, staining only sparse cells outside the tubules in 5 ABCs. This possibly explains 100% positivity rate in another study [4], as 2 of our ABCs did not contain well defined glandular structures. Positivity rate of NKX3.1 and HoxB13 was comparable in SCCs and EACs and represented 9.5% and 21.1% respectively. HoxB13 was generally stronger, with IRS 8 in one SCC and IRS 4 in one EAC. However, no NKX3.1 was observed in all grade 3 ECs, in contrast to 40% positivity rate for HoxB13 (IRS 1–2). Thus, any positivity of HoxB13 was 80% specific and 100% sensitive for the ABC. NKX3.1 was only 82.4% sensitive, but 88% specific for the diagnosis of ABC. On the other hand, 46.7% of ABCs showed strong and diffuse HoxB13 (IRS 12) and additional 20% showed IRS ≥ 6, contrasting with generally weak immunoreactivity in other carcinomas (IRS 1–2). Therefore, strong and diffuse HoxB13 is highly uncommon beyond ABC. HoxB13 was also significantly more often seen in HSILs adjacent to ABCs. Given the fact that ABCs are HPV-related tumors, this suggests that ABC phenotype may be predetermined to some extent already in non-invasive stages of the disease.

As for the significance of HoxB13 in cervical lesions, it must be emphasized that we also observed a common immunoreactivity of HoxB13 but not NKX3.1 in the basal layer of morphologically normal vaginal and cervical squamous epithelium around the studied lesions. Thus, HoxB13 seems to rather reflect spatial identity of the cells in this location than bona fide prostatic differentiation. The pattern of expression of both markers also differed, as NKX3.1 positivity was restricted predominantly to the glandular structures, similar to the previous report [3]. On the other hand, HoxB13 was also positive in squamous/basaloid epithelium in majority of EPTs, TSPs, and ABCs. This is not surprising, given the AR-dependent expression of NKX3.1 in luminal cells of the prostate and the role of NKX3.1 in luminal cell differentiation [8, 9]. In contrast, weak immunoreactivity of HoxB13 has been reported in basal cells of the prostate and we also observed weak expression (IRS 2 to 3) in all analyzed cases of prostatic basal cell hyperplasia. Interestingly, AR was observed only in 26.3% (5/19) of TSPs and EPTs and no AR was detected in any of ABCs. This suggest that the transcriptional regulation of the prostatic phenotype differs between gynecological tract entities and normal prostate. In our opinion, it is possible that HPV-related oncogenesis triggers a prostatic-like differentiation in permissive cervical epithelium that shares developmental analogy with prostate. The lack of true prostatic differentiation would be further supported by the absence of any reported ABCs arising in EPT or vaginal TSP as well as by the complete lack of AR in our ABC cohort, in contrast to occasional positivity in TSPs and EPTs.

In conclusion, both HoxB13 and NKX3.1 are expressed in TSPs, EPTs and ABCs, with HoxB13 showing stronger overall immunoreactivity and improved sensitivity, with slightly lower specificity (80% vs 88%) compared to NKX3.1. On the other hand, NKX3.1 is more useful for identification of glandular structures. ABCs are consistently p16-positive, suggesting HPV-related carcinogenesis, while ABHs lack p16 or prostatic transcription factors and this supports that ABH is nosologically unrelated lesion without malignant potential. Furthermore, combination of p16 and HoxB13 can be utilized to distinguish ABC and ABH in limited samples.

## Supplementary Information

Below is the link to the electronic supplementary material.

Supplementary file1 (XLSX 24 KB)

## Data Availability

The immunohistochemical results of individual tumors, together with basic clinical data, are available as supplementary material. Additional clinical data and histological images (in the form of ‘*.svs’ formatted image files) used and analysed during the current study are available from the corresponding author upon reasonable request.
